# Clinical Psychological Figures in Healthcare Professionals: Resilience and Maladjustment as the “Cost of Care”

**DOI:** 10.3389/fpsyg.2020.607783

**Published:** 2020-12-01

**Authors:** Emanuele Maria Merlo, Anca Pantea Stoian, Ion G. Motofei, Salvatore Settineri

**Affiliations:** ^1^Department of Cognitive Sciences, Psychology, Educational and Cultural Studies (COSPECS), University of Messina, Messina, Italy; ^2^CRISCAT (International Research Center for Theoretical and Applied Cognitive Sciences), University of Messina and Universitary Consortium of Eastern Mediterranean, Noto (CUMO), Noto, Italy; ^3^Faculty of General Medicine, Carol Davila University of Medicine and Pharmacy, Bucharest, Romania; ^4^National Institute of Diabetes, Nutrition and Metabolic Diseases “N. C. Paulescu,” Bucharest, Romania; ^5^Department of Biomedical and Dental Sciences and Morphofunctional Imaging, University of Messina, Messina, Italy

**Keywords:** healthcare professionals, caregivers, burden, clinical psychology, resilience (psychological)

## Abstract

**Background:** The health professionals are involved in the paths of care for patients with different medical conditions. Their life is frequently characterized by psychopathological outcomes so that it is possible to identify consistent burdens. Besides the possibility to develop pathological outcomes, some protective factors such as resilience play a fundamental role in facilitating the adaptation process and the management of maladaptive patterns. Personal characteristics and specific indexes such as burdens and resilience are essential variables useful to study in-depth ongoing conditions and possible interventions. The study was aimed at highlighting the presence and the relations among factors as personal variables, burdens, and resilience, to understand health professionals' specific structure and functions.

**Methods:** The observation group was composed of 210 participants, 55 males (26.2%), and 155 females (73.8%), aged from 18 to 30 years old with a mean age of 25.92 years old (*SD* = 3.33). The study considered personal characteristics of the subjects, such as age, gender, years of study, days of work per week, hours of work per week, and years of work. Our study had been conducted with the use of measures related to burdens (Caregivers Burden Inventory) and resilience (Resilience Scale for Adults).

**Results:** The performed analyses consisted of descriptive statistics, correlations, and regressions among the considered variables. Several significant correlations emerged among personal characteristics, CBI, and RSA variables. Specifically, age and work commitment indexes appeared to be significantly related to the development of burdens, differently from the years of study. Significant correlations emerged among personal and RSA variables, indicating precise directions for both domains. Age and gender were identified as predictors to perform multivariate regression analyses concerning CBI factors. Significant dependence relations emerged with reference to all CBI variables.

**Conclusion:** Pathological outcomes and resilience factors represent two sides of the health professionals' experiences, also known as “invisible patients.” Greater knowledge about present conditions and future possibilities is a well-known need in literature so that the current analyses considered fundamental factors. In line with state of the art, future studies are needed in order to deepen elusive phenomena underlying maladjustment.

## Introduction

The healthcare professions expose the subjects to different pathological dynamics, which may retroactively refer to them depending on working conditions and clinical issues. In general, many contributions in literature highlighted how the health status of caregivers meets different challenges, of physical, mental, and management order, extended to their personal life up to the threshold of mortality (Schulz and Beach, 1999; Von Känel et al., 2003; Aschbacher et al., 2006; Kannan et al., 2011; Iavarone et al., 2014). The personal characteristics of these “invisible patients” (Bevans and Sternberg, 2012; Adelman et al., 2014) represent relevant variables since the burdens they have to bear are always relative to the intersection of personal and professional life.

The risks are well-known and well-presented in the literature; for 30 years, many classic studies considered the main variables of the subjects assisting patients with heterogeneous pathological conditions and adverse experiences (Provencher, 1996; Greenberg et al., 1997; Shdaifat and Manaf, 2012; Nikmanesh and Shahinfar, 2016; Di Giuseppe et al., 2018, Di Giuseppe et al., 2019b; Guicciardi et al., 2019; Marchi et al., 2019). In a previous article, it was highlighted how health professionals could meet diametrically opposed dynamics presenting strengths and significant vulnerabilities (Settineri et al., 2019a, Settineri et al., 2019c). The relationships emerged among variables such as well-being, compassion, burnout, and emotional state, pointed out the possible adverse outcomes that caregivers face.

Specifically, constant contact with pathological realities can produce maladjustment from both a professional and health point of view. Dysphoric polarities and low levels of well-being emerged as dominant traits, along with dynamics of compassion and strengths.

What dominates the literature on the caregivers' theme is the need to implement the knowledge about the phenomena and the solution of the pathological outcomes of caregivers.

Similarly, Merlo et al. (2020a) highlighted two precise faces about the profession of caregiver, respectively, referring to compassion and suppression of disturbing contents that tend to be transported into their private life. The impact of these operations on the community is fundamental. Still, it cannot be too far from the personal dynamics of the caregivers who always have to solve the existing ambivalence between compassion satisfaction and fatigue (Hadjistavropoulos et al., 1994; Hundall Stamm, 2009; Collins and Swartz, 2011; Makic, 2015; Velutti et al., 2017; Lynch et al., 2018; Wood et al., 2018; Settineri et al., 2019f; Allday et al., 2020).

A large amount of scientific contributions has dealt with caregivers in general terms. A particular category of caregivers in terms of age is that of the subjects at the beginning of their career. Despite the prevalence of health professionals who practice this profession who are of adulthood, there are many cases in which young professional caregivers could immediately begin to experience the pathological conditions of the patients (Hawken et al., 2019).

In particular, the dynamics taken into consideration in this study referred to specific possibilities and outcomes. First of all, the issue is relating to the various types of caregivers' burden.

Previous articles considered the concept of the burden since caregivers presented a decreased quality of life and specific relationships to the pathological conditions of the assisted subjects (Shdaifat and Manaf, 2012; Grant et al., 2013; Nikmanesh and Shahinfar, 2016; Widowati et al., 2018). The concept of burden clearly represents a homogeneous set of phenomena present in the entire population, as stated by Chou (2000) in his conceptual analysis of the involved dynamics.

As reported by Liang et al. (2018), several studies highlighted predictors and risk factors of this profession, including female gender (Gallicchio et al., 2002), low education (Gallagher et al., 1989; Vincent et al., 2009; Wong et al., 2012; Adelman et al., 2014; Cole et al., 2014), sleep deprivation, economic distress, mood disorders, isolation, and different material restrictions.

Among the possible experiences, the high probability of developing adverse outcomes in psychopathological terms is well-known. This is not the only choice, since we are witnessing the presence of adaptation conditions that respond to the logic of compassion satisfaction. Compassion satisfaction represents a protective factor with respect to the negative possibilities hitherto treated. Specifically, some contributions in literature allowed us to evaluate its role together with work activities (Stamm, 2002; Hundall Stamm, 2009; Collins and Swartz, 2011; Makic, 2015; Lynch et al., 2018; Allday et al., 2020).

In general, this ambivalence testifies to the fact that caregivers and healthcare professionals must be in a position of resistance to those phenomena bringing them closer to negative outcomes. The concept of resilience first appeared in 1992 (Werner and Smith, 1992; Peveri, 2009) as the first example of a clear conceptualization of the phenomenon.

The origin of the term is of a different domain since, in physics, it represents the attitude of a body to resist without breakage following abrupt or lasting external mechanical stresses (Devoto, 1971).

The conceptualization they introduced into the domain of the psychological disciplines has dealt with the neuroscientific and psychobiological side, with particular reference to traumatic experiences (Edelman, 1992; Damasio, 1994; Le Doux, 1996; Cyrulnik and Malaguti, 2005; Malaguti, 2005; Putton and Fortugno, 2006; Motofei and Rowland, 2015, 2016, 2018).

Resilience, based on various contributions in the literature (Rutter, 1987, Rutter, 1990, Flach, 1988; Fine, 1991; Garmezy, 1993; Rutter, 2007), represents a dynamic phenomenon, which, depending on the environmental conditions, leads the subject to get closer to adaptation.

An integrative contribution (Richardson, 2002) brought together the existing approaches, essentially distinguishing two research phases and coming to the conclusion that resilience factors are present since birth and can be differently enhanced during the life span (Peveri, 2009).

Recent research papers (Harmell et al., 2011; Fernández-Lansac et al., 2012; Lin et al., 2013; Dias et al., 2015) continue to confirm the centrality of resilience with respect to the adaptation possibilities of caregivers. Among these, a review according to the conceptual aspects of the term resilience highlighted that various factors contribute to the caregivers' well-being (Lin et al., 2013).

Factors such as disposition patterns, situational patterns, relational patterns, and cultural patterns represented the dimensional constructs of resilience for caregivers working with children experiencing the chronic condition. Harmell et al. (2011) reported the higher levels of personal mastery and self-efficacy of caregivers as the main finding, showing their capacities in the light of protective issues.

According to Chapman et al. (2020), some fundamental frameworks need to be considered about resilience. Some contributions treated by the authors exemplified this need as pointed out through the interest for the adverse events (Bonanno, 2004) and the related maladaptive outcomes (McVicar, 2003).

With particular reference to Wright et al. (2013), the authors reported the definition of an adverse event as: “*disturbances to the function or viability of a system”* (p. 17). It is clear that the theme of adversity extends from very small systems to systems of enormous scope and that several components are playing a fundamental role, such as time (Bonanno et al., 2011, 2015; Cosco et al., 2017). Based on the figures treated so far and the possible implications related to their interaction, four hypotheses were formulated in order to research the precise outcomes. The details of these formulations and the potential implications have been reported in the following paragraph.

### The Current Study

The aim of this study was to highlight the role of different factors, such as personal caregivers' characteristics that are included in the Caregivers Burden Inventory and Resilience Scale for Adults. In order to highlight correlations and dependence relations among the factors mentioned above, the following hypotheses stated:

Hp-1: We hypothesize that the caregivers' personal characteristics (age, years of study, days of work per week, hours of work per week, and years of work) are significantly correlated to CBI factors (time dependence burden, developmental burden, physical burden, social burden, emotional burden, and CBI total score). In particular, we hypothesize positive (+) relations among all factors, specifically years of study characteristic, known as a protective factor in terms of possible burden outcomes.

Hp-2: We hypothesize that the caregivers' characteristics (age, years of study, says of work per week, hours of work per week, and years of work) are significantly correlated to RSA factors (perception of self, planned future, social competence, structured style, family cohesion, social resources, and RSA Total score), considering the central role of resilience for healthcare professionals and the weight of long time assistance activities.

Hp-3: We hypothesize that the CBI factors (time dependence burden, developmental burden, physical burden, social burden, emotional burden, and CBI total score) are significantly correlated to RSA factors (perception of Self, planned future, social competence, structured style, family cohesion, social resources, and RSA Total score) with a particular reference to the possible distinctions among conceptual differences and the role of time in the caregivers' experiences.

Hp-4: We hypothesize the existence of significant dependencies with reference to the two selected predictors, namely, age and gender, and CBI factors (time dependence burden, developmental burden, physical burden, social burden, emotional burden, and CBI total score), highlighting the causal role of age and gender in developing burdens related to health professionals.

## Materials and Methods

### Procedure and Participants

The observation group consisted of 210 participants, 55 males (26,2%) and 155 females (73,8%). The participants' age included in the study was between 18 and 30 years old, with a mean age of 25.92 years old (*SD* = 3.33).

The research was carried out in the University Hospital G. Martino of Messina. The subjects were selected on the basis of their work activities. It has been possible to involve 210 caregivers in order to administrate the complete protocol. Every participant fully completed the questionnaires, including information regarding their work activity, gender, and age.

The compilation of the questionnaires was in online form. Each participant, before adhering to informed consent, was informed about the anonymous nature of the methods of data processing as required by the procedures of the ethical committee evidenced by the approval (University of Messina COSPECS Ethical Committee, Ethical committee number: COSPECS_11_2020).

### Statistical Analysis

The data were expressed as mean and standard deviation and the categorical variables as number and percentage.

The “Spearman test” was applied in order to evaluate the correlations among variables of the following instruments.

Multivariate linear regression was used to assess the dependence of each burden-related outcome (time dependence, developmental burden, physical burden, social burden, emotional burden, and Total CBI score) on a set of independent predictors (age and gender).

Statistical analyses were performed using the SPSS 26.0 for Windows package.

A *P*-value smaller than 0.050 was considered to be statistically significant.

### Instruments

#### Caregivers Burden Inventory

The “Italian version of the Caregiver Burden Inventory” (CBI) (Novak and Guest, 1989), a 24-item multidimensional questionnaire that measures the caregiver's burdens along five dimensions: time dependence, developmental, physical, social, and emotional, was evaluated using a “five-point Likert” scale from 0 (not at all disruptive) to 4 (very disruptive). Several studies examined the reliability both for the total scale and the different factors. In particular, in a recent study by Greco et al. (2017), the CBI was administrated to a sample of Italian caregivers. In the study, the Cronbach's alphas were, respectively, 0.96 for the total scale, 0.91 for time dependence, 0.92 for developmental burden, 0.88, 0.89, and 0.93 for physical, social, and emotional burden. In a second study published by Conti et al. (2019), the total score index was 0.91 and ranging from 0.76 to 0.91 for the abovementioned factors.

#### Resilience Scale for Adults

The Resilience Scale for Adults (RSA) is a self-report scale based on 33 items, measuring the protective factors related to resilience in adults (Hjemdal et al., 2001; Friborg et al., 2003, 2005; Capanna et al., 2015). As reported by Bonfiglio et al. (2016). The original structure of the RSA includes six factors related to both personal (personal strength, planned future, social competence, and structured style) and external factors (Friborg et al., 2005; Hjemdal et al., 2006; family cohesion and social resources). As reported by Hjemdal et al. (2001), the final version presents a six-factor solution including factors named: (1) Perception of self (Cronbach's α = 0.74), (2) Planned future (α = 0.73), (3) Social competence (α = 0.83), (4) Structured style (α = 0.80), (5) Family cohesion (α = 0.80), and (6) Social resources (α = 0.74). The alphas of the total scale were ranged between 0.82 and 0.95, including the original version and the subsequent adaptations (Friborg et al., 2005; Hjemdal et al., 2006).

## Results

Descriptive statistics (the mean and the standard deviation) are reported in Table 1, in order to highlight the presence of the considered phenomena.

**Table 1:** Descriptive statistics for study variables.

	**Mean**	**Standard deviation**
Years of study	14.94	2.69
Days of work per week	5.34	1.17
Hours of work per week	36.47	19.22
Years of work	3.68	3.97
Time dependence burden	9.16	5.49
Developmental burden	5.58	5.17
Physical burden	4.44	3.82
Social burden	3.90	4.34
Emotional burden	2.68	4.27
CBI total score	25.77	16.98
Perception of self	17.89	2.46
Planned future	12.14	2.09
Social competence	17.62	2.43
Structured style	11.79	1.87
Family cohesion	18.93	2.90
Social resources	19.30	2.72
RSA total score	97.68	8., 56

### Hypothesis 1

Hypothesis 1 concerned the relationships between subjects' work variables and different types of burden. The significant associations between the personal components and the maladaptive outcomes of the different types of burden were hypothesized (see Table 2).

**Table 2:** Correlation coefficients among personal-work variables and CBI factors.

	**Age**	**Years of study**	**Days of work per week**	**Hours of work per week**	**Years of work**
Time dependence burden	**0**.237^**^	**−0**.179^**^	**0**.182^**^	0.091	0.093
Developmental burden	**0**.190^**^	**−0**.164^*^	**0**.224^**^	**0**.160^*^	**0**.225^**^
Physical burden	**0**.163^*^	**−0**.**072**	0.068	0.091	0.107
Social burden	−0.030	**−0**.151^*^	0.088	0.084	0.079
Emotional burden	−0.046	−0.082	−0.079	0.009	−0.040
CBI total score	**0**.153^*^	**−0**.209^**^	**0**.173^*^	0.129	**0**.138^*^

* *p < 0.05 (two-tailed)*.

** *p < 0.01 (two-tailed)*.

*Bold values were the significant values*.

In particular, the correlation analysis included age, years of study, days of work per week, hours of work per week, and years of work (total as a caregiver) as caregivers' work variables. In terms of burden, the values of each individual burden type were considered, as time dependence, developmental, physical, social, and emotional burden, and total CBI score.

Going in order, the first personal variable considered was age. Several significant correlations emerged. A significant and positive relationship emerged between age and time dependence, showing how advancing age had the same direction of developing temporal difficulties. Precisely, the direction of increasing age corresponded to an increase in temporal burden. Similarly, age indicated its increase as corresponding to the significant rise in developmental and physical burdens. The three components were significantly and positively correlated with age. Finally, the total score expressed the same direction as the already mentioned components. A significant and positive correlation emerged between age and the total score of the Caregivers Burden Inventory.

Years of study of caregivers represent the second personal variable taken into consideration. It is possible to immediately notice that the years of study could represent a protection factor for the caregivers since all the significant correlations were of a positive sign. This applied to time dependence, developmental burden, social burden, and the CBI total score. These newly expressed relationships showed significant and negative directions; therefore, as the years of caregivers' study grew, there was a decrease in the single burden scores and the total score.

In line with what emerged for age, the variables “days of work per week,” “hours of work per week,” and “years of work” showed significant and positive relationships with the burdens (single factors and total score). Concerning the first factor, the meaningful relationships of days of work per week were those with time dependence, developmental burden, and with the total score (CBI total score). The number of working days per week increased; there were higher scores in terms of time-related burden, developmental burden, and in general terms (CBI total score). The significant relationship for working hours was with developmental burden, demonstrated by how many hours of work per week corresponded to the sense of failure of caregivers' personal prerogatives.

In terms of years of work, significant relationships with the developmental burden and the total score were found. The interesting data emerged in systematic terms, referred precisely as developmental burden, that pathological outcome corresponds to the sense of failure of one's personal hopes and life expectations. The general trend was, in fact, positive with all variables, except with years of study (already meant as a protective factor).

As already clarified, it appeared that the years of education and study of the caregivers corresponded to an opposite direction of burden development. In the case of the sense of failure for personal ambitions, however, the data are indicative of the positive relationship between the increase in work commitment and maladaptive outcomes.

### Hypothesis 2

Hypothesis 2 and as shown in Table 3, several significant correlations were found among caregivers' personal variables and resilience factors. Starting from age, three negative and significant correlations emerged, respectively, with perception of self, structured style, and family cohesion.

**Table 3:** Correlation coefficients among personal-work variables and RSA factors.

	**Age**	**Years of study**	**Days of work per week**	**Hours of work per week**	**Years of work**
Perception of self	**−0**.115^**^	**0**.097^*^	**−0**.106^**^	−0.038	−**0**.092^*^
Planned future	**0**.105^**^	−0.057	**0**.205^**^	**0**.100^**^	0.037
Social competence	0.041	−0.025	0.041	0.037	−0.023
Structured style	**−0**.092^*^	−0.082^*^	−0.037	−0.015	−**0**.137^**^
Family cohesion	**−0**.087^*^	**0**.081^*^	−0.013	0.020	−0.029
Social resources	0.065	−0.061	**0**.127^**^	−0.001	−0.005
RSA total score	−0.012	−0.010	**0**.084^*^	0.059	−0.053

* *p < 0.05 (two-tailed)*.

** *p < 0.01 (two-tailed)*.

*Bold values were the significant values*.

These relations indicated that the increase in age corresponded to a decrease in the general perceived confidence about subjects' efficacy, abilities, and positive outlook. In the same direction, the other two negative relations pointed out the decrease of a structured style and family cohesion, indicating goal-orientated activities and shared family values, enjoyment of situations, and a collective optimism for family-orientated perspectives.

The positive correlation that emerged as significant was with planned future, highlighting a growing sense of belief about possible opportunities and future perspectives. Years of the study appeared to be in a positive relationship with both perception of self and family cohesion, indicating a common direction of the years of education with the general self-perceived range of abilities and possibilities, together with enjoyable family figures.

Days of work per week showed a significant and positive relationship with planned future, social resources, and RSA total score, indicating similar orientations. Contrary to this latter point, the perception of self emerged in inverse relation, so that caregivers' personal beliefs about self-efficacy and abilities seemed to decrease in relation to the increase in days of work.

Hours of work per week showed a significant and positive relationship with planned future, while years of work had two inverse and significant correlations with perception of self and structured style, to properly mark the impact of a long work commitment on self-efficacy and planned activities.

### Hypothesis 3

As shown in Table 4, positive correlations were found among CBI and RSA variables, with a particular reference to two strong domains, namely, planned future and social resources. The first reference is due to the perception of self, showing two significant and inverse correlations with developmental burden and the Caregiver's Burden Inventory total score. In detail, the prerogatives related to the caregiver's sense of self-efficacy emerged as inverted with the possibility to develop an understanding of failure linked to personal hopes and ambitions. The general score indicated the same direction as that of self-efficacy, and personal representations about general and specific abilities show themselves as opposite to burdens and vice versa. Planned future was involved in several and interesting relations, all positive and significant. All CBI factors emerged indicating directions with positive outlooks, beliefs, and the necessity to plan for the future. Social resources were in positive and significant correlations, respectively, with time dependence burden, developmental burden, physical Burden, and the CBI total score.

**Table 4:** Correlation coefficients among CBI and RSA variables.

	**Perception of**	**Planned**	**Social**	**Structured**	**Family**	**Social**	**RSA total**
	**self**	**future**	**competence**	**style**	**cohesion**	**resources**	**score**
Time dependence burden	−0.068	**0**.174^**^	−0.002	−0.074	−0.074	**0**.089^*^	0.014
Developmental burden	**−0**.117^**^	**0**.308^**^	0.047	−0.005	0.002	**0**.130^**^	**0**.118^**^
Physical burden	−0.064	**0**.300^**^	0.054	−0.056	−0.028	**0**.093^*^	**0**.083^*^
Social burden	−0.059	**0**.205^**^	−0.043	0.009	−0.053	0.026	0.060
Emotional burden	−0.040	**0**.155^**^	0.070	0.006	−0.001	0.014	0.067
CBI total score	−**0**.099^*^	**0**.297^**^	0.028	−0.037	−0.037	**0**.102^**^	**0**.085^*^

* *p < 0.05 (two-tailed)*.

** *p < 0.01 (two-tailed)*.

*Bold values were the significant values*.

### Hypothesis 4

To study dependence relations, as reported in Table 5, age and gender effects were involved as independent variables and predictors in their relationships with CBI variables. As emerged through the analysis, significant causal relations emerged among variables. About age, three relations were highlighted, respectively, with time dependence, developmental burden, and physical burden. These relations explained the relevant role of age in increasing the possibility to experience burdens. Going in-depth, the burden depending on the time dedicated to clinical assistance resulted in being predictable as a final outcome due to aging, as also for caregivers' belief to be cut off from possible life's opportunities and the presence of physical outcomes. Regarding the effect of gender, significant dependencies were clearly pointed out by the regression analysis, in particular among gender and developmental, social, emotional burden, and CBI total score. Male gender appeared to be a significant predictor of the decrease in the already mentioned outcomes so that all significant relationships were negative. These data referred to the limitations coming from the excessive assistance activities, social and emotional outcomes, and the general possibility to develop burden.

**Table 5:** Multivariate linear regressions analysis.

	**Age**		**Gender**	
	**B (CI)**	***P***	**B (CI)**	***p***
Time dependence burden	0.377 (0.147/0.608)	0.001^*^	−0.,202 (−1.872/1.468)	0.812
Developmental burden	0.241 (0.032/0.451)	0.024^*^	−1.997 (−3.517/−0.477)	0.010^*^
Physical burden	0.177 (0.014/0.339)	0.033^*^	−0.858 (−2.035/0.320)	0.152
Social burden	−0.047 (−0.230/0.136)	0.614	−2.230 (−3,554/−0.906)	0.001^*^
Emotional burden	−0.140 (−0.324/0.044)	0.134	−1.847 (−3,180/−0.514)	0.007^*^
CBI total score	0.608 (−0.098/1.315)	0.091	−7.133 (−12.250/−2.016)	0.007^*^

*B, beta coefficient; CI, confidence interval*.

* *p < 0.05 was considered as significant for the multivariate linear regression analyses*.

## Discussions

Our study was aimed at highlighting the existing relations among different factors, such as the personal characteristics of the caregivers and the variables referred to as burdens and resilience. In the various sections of the results, different hypotheses have been presented, and the analysis has highlighted many significant relationships. In particular, the study showed two classes of research, oriented to the detection of correlations and causality relationships. The comparison between the caregivers' characteristics and the factors of the reference domains, represented a useful occasion for the comparison between the emerged results, current, and previous literature. The analysis, therefore, considered factors such as age and gender of the caregivers, together with their attitude to work (in a temporal sense) and educational years. The choice to compare these variables with those of the abovementioned domains mainly depended on the fact that the courses related to these healthcare professions were activated in our university.

This university opening is part of the logic that various authors followed regarding clinical efforts. With particular reference to clinical practice, our orientation corresponds to the current needs and challenges in the field of clinical psychology (Caputo, 2013; Carrozzino et al., 2019; Conversano, 2019; Martino et al., 2019b; Merlo, 2019a,b; Settineri et al., 2019d). The results obtained through the analysis, starting from the question of burden, highlighted some fundamental facts. The age of the caregivers always explained positive directions with the burdens identifying specific issues. What appeared opposed to the increase in the age of the caregivers was precisely the ability to plan the future and manage time, which refers to the personal life of the caregivers. The same directions were taken by the developmental burden, and therefore addressed to personal ambitions and possibilities of self-realization and extended to physical and general issues.

As stated by Win et al. (2017), according to Lim et al. (2014), some data emerge in close relationship with the burden in the field of performance. In both contributions, the authors noted changes in the caregivers' performance with reference to age. The first contribution cited statistically ascertained significant differences, suggesting the central role of age with reference to worrying feelings. All of these clearly affect the performance of healthcare professionals. If, on the one hand, the younger caregivers experience the feeling of worry about work activities, on the other hand, the older caregivers instead experience high levels of burden and fatigue.

This datum could be extended to other variables considered in terms of work commitment, therefore the years of study, hours, and days of work per week. The significant relationships highlighted congruent directions between the development of the burdens and the increase in work commitment. As supported by Stanley et al. (2017), the outcomes of exposure to difficult existential conditions bring outcomes to the mental and physical health of caregivers (Mitsonis et al., 2012; Suro and Weisman de Mamani, 2013; Gater et al., 2014; Gupta et al., 2015; Stanley et al., 2016). The literature just mentioned opens the dialogue about the issue of interventions and education of caregivers. We referred to specific educational programs for university courses.

Our results revealed a significant and negative correlation between the years of study of the caregivers and the different types of burden, including time dependence, developmental burden, social burden, and the overall scores. The occupational and educational question of caregivers has long been discussed (Toth-Cohen, 2000), and several authors treated the need for specific educational and support programs for caregivers and family members (Ji et al., 2014). The result to which we refer indicated a decrease in the development of the aforementioned burdens, followed by the increase in the years of education. This result was in line with recent scientific contributions (Greene and Monahan, 1989; Lee and Kim, 2017; Mukherjee, 2017; Batra et al., 2018; Cianfrocca et al., 2018; Conant, 2019; Hekmatpou et al., 2019).

In terms of risk and protection factors, it is well-known that different personal and environmental characteristics affect the life of the healthcare professionals (Haley et al., 2003; Lovell et al., 2012a,b; Dardas and Ahmad, 2014; Bekhet and Matel-Anderson, 2017). Among the various factors involved, according to the current state of the art, it is possible to identify particularly adaptive trends for caregivers (Pagnini et al., 2016), which allowed us to consider basilar factors as in the case of resilience. According to Palacio et al. (2018), factors such as resilience, perceived competence, emotional regulation, and positive aspects of care represent fundamental points for the caregivers' health conservation.

In terms of resilience, our results highlighted specific trends, so that starting with the age increase in the caregivers, there was a decrease in self-perceptions, adaptive styles, and family cohesion. Conversely, qualities such as planning for the future appeared to be inversely related. The years of education and professional training took directions akin to the ability to remain adherent to an adaptive self-perception and a cohesive family. The variables related to work commitment in the week, in the month, and in general had several significant correlations. In detail, the perception of self always appeared negatively associated with variables, but rather for years of study. The overall years of work showed a negative relationship with the structured style, indicative of a decrease in containment and coping capacity. Several studies have been previously mentioned, indicating the relationships between variables related to burdens experienced by caregivers and resilience factors. In our results, the relationships that immediately appeared strongly correlated concerned the relationship of planned future with all burdens. Positive and significant relationships emerged, certainly guaranteeing to understand the temporal dynamics of the caregivers.

Several studies previously mentioned indicated the relationships between variables related to burdens experienced by caregivers and resilience factors. In our results, the relationships that immediately appeared strongly concerned about the relationship of the planned future variable with all burdens. Positive and significant relationships emerged, certainly guaranteeing the comprehension with reference to the temporal dynamics of the caregivers. The “cost of caring” is a concept that manifests itself through issues on future planning (Wang and Han, 2019), with greater prevalence in the caregivers of patients with particularly serious medical conditions. In the study of the factors affecting these limitations, Walker and Hutchinson (2018) reported the establishment of some barriers that prevented future planning for caregivers. Although the need was apparent, intervening factors appeared exactly as barriers.

The authors reported fundamental studies about these barriers, such as that of Davys and Haigh (2008) indicating the presence of a planning gap due to internal psychological figures. In the same direction, Dillenburger and McKerr (2011) and Stehlik (2000) highlighted the fact of feelings of helplessness, resignation, avoidance, denial, and guilt about not planning. A second relevant dimension was that of uncertainty, so the authors reporting previous articles (Eley et al., 2009; Pryce et al., 2017) showed how the subjects withdrew from the planning phase due to this additive burden. The contribution of Walker and Hutchinson (2018) represents an example of practical analysis, which allows us to understand how relevant the temporal issue is in the life span. Regarding the emergence of other significant relationships, the intersection of the variables of the two scales highlighted positive directions between the burden and the social dynamics of the caregivers. A metanalysis by Parker Oliver et al. (2017) on the social issue and the work commitment of caregivers highlighted fundamental phenomena. Social isolation and the incidence of burdens is usually widespread, and psychosocial outcomes occur at different levels, mental, physical, and social.

The last hypothesis concerned the level of causal incidence of some predictors on burden phenomena. Significant causal relationships identified a primary role for the two predictors, as several results emerged. Age and gender were related significantly to the burdens, to the point that none of the individual burden was excluded from significant relationships. In detail, age had an incident role on the temporal, developmental, and physical burden. Gender was in a relationship of significant dependence with the developmental, social, and emotional burden. The overall score was in a dependence relation with the overall burden factor. In line with some contributions in the literature, the results obtained showed a clear impact of these two dynamics on the possibility of developing burdens in caregivers.

What emerged from the results of this work, referred to the presence of different dynamics, which, respectively, consisted of adverse outcomes due to clinical care and the ability of caregivers to cope with difficulties. The subject of burdens has been treated by many contributions in the literature, some of which have proposed different types of intervention. It is necessary to understand that among the various types of existing caregivers, duly educated professional figures are flanked by informal caregivers. In most cases, caregivers take on the role of family members or relatives who take responsibility for care. Future interventions and perspectives must take account of these dynamics, right from the early educational stages of professionals.

Many of the caregivers have not received specialized education, to the point that they do not have skills useful for dealing with the pathological realities of patients. This figure is also extended to those professional figures who by definition acquire skills during the university training courses (Langher et al., 2014, 2018; Parola and Donsì, 2018, Settineri et al., 2018; Parola and Donsì, 2019; Merlo et al., 2020b; Parola and Felaco, 2020). The success or failure of therapies and treatments often depends on the skills of health professionals, and this suggests that they cannot be overlooked, with particular reference to adult and adolescent medical conditions (Mullen, 1997; Brown, 1999; Lingiardi et al., 2010; Shrivastava et al., 2013; Sugiharto et al., 2017; Di Giuseppe et al., 2019a; Martino et al., 2019a; Rosa et al., 2019, Settineri et al., 2019e; Ardeleanu et al., 2020; Martino et al., 2020; Moroianu et al., 2020; Muzi, 2020; Nedelcu et al., 2020).

Regarding the commitment of informal caregivers, several authors have conducted analyses that, despite the past years, remain effective and well-structured (Yates et al., 1999; Donelan et al., 2002; Pinquart and Sörensen, 2007). As already stated, the research results represent the basis on which the necessary interventions should arise, although educational practices must describe the first step. The fact that these educational gaps currently persist places caregivers in a position to experience adverse outcomes even today (Benson et al., 2020; Campione and Zebrak, 2020; Clancy et al., 2020; Price et al., 2020). The consequences relating to the non-observation of these personal, educational, and institutional needs are manifold and extend even to the defensive, neuropsychological, and neurovegetative sphere (Schredl, 2013; Dell'Osso et al., 2014; Rania et al., 2018; Catalano et al., 2019; De Stasio et al., 2019; Schredl et al., 2019; Settineri et al., 2019b; Di Giuseppe et al., 2020a,b; Hoyt et al., 2020; Romero-Martínez et al., 2020; Somma et al., 2020).

Our experience involved young caregivers, whose commitment in the beginning produce dysfunctional outcomes since the early years of work. This fact highlights two central points, respectively, the need for intervention and the implementation of educational programs for all professionals. One of the merits of this type of research consists in the possibility of explaining the origin and the existing relationships between phenomena that are noticed through valid instruments. Further contributions should take into account a different but, nonetheless, central level with regard to the expression of health and pathological conditions experienced by the caregivers. The potential and function of the narrative of the subjects (Bourlot, 2018, 2020) represent relevant figures in clinical relationships.

The expressive modalities of the subjects are conditioned by approaches which, despite guaranteeing an excellent statistical and population framework, neglect the subjective aspects through which the therapeutic process is carried out.

A substantial set of contents could emerge through narrative analyses, which in most cases escape statistical frameworks and which are expressed through manifestations that mask the real meanings (Bourlot, 2019; Manfredi and Massardi, 2019; Settineri et al., 2019d). The advantage made possible by the current analysis refers to the production of evidence that testifies to general conduct, which compared to the other works present in the literature allows the researchers to orient the resolutive plans of the conditions currently current. In our experience, in fact, the difficulties witnessed by the presence of burdens were accompanied by attitudes of resilience and followed by adaptive and compensatory perspectives. In the future, it will be necessary to envisage variations and changes that comply with the evidence that emerged from the literature and from the commitment of clinicians to the health of patients and those “invisible patients” who dedicate themselves every day to a very particular type of assistance.

## Implications of the Study

In order to highlight relevant dynamics occurring in health professional's experience, two important factors were taken into account. Health professionals and caregivers represent an important category, reflecting unknown phenomena of health sectors. The analyses were based on the selected themes in order to compare well-known dynamics with outcomes related to work. Some considerable results emerged, suggesting how independent factors such as age and gender impact caregivers' professional and private life. The emerged data made it possible to compare our results with previous published research evidence. In these terms, it was possible to make it clear as well-known dynamics were also present in our territory.

The choice to maintain the metaphor “invisible patients” depended on a lack of knowledge on caregivers' difficulties. Our results highlighted a continuity with those contributions that showed the need for a greater knowledge on the treated phenomena. The aim to distinguish the negative outcomes from the positive possibilities, such as resilience, was due to the need to point out adaptive strategies related to prevention and support. The results that emerged can be considered as evidence on the basis of which future interventions may arise.

## Limitations and Conclusion

This study presents a diversity of limitations that should be overcome in future studies. The number of health professionals employed in public and private realities is high, and subsequent studies should expand the observation group. This would lead researchers to the possibility of extending the data to a more representative population. The number of participants has an evident prevalence of female subjects, and although it is known that the population of caregivers in the area is predominantly female, a gender balance would be necessary. The reference to young caregivers would require more studies to support their relevance and the negative outcomes experienced. Although this latter reference constitutes a limitation, this study was aimed at implementing knowledge about it. The relevant figure was included by the presence of burdens from the early years of experience of the caregivers, accompanied by good resilience skills. This fact can be considered a guarantee of the understanding of the need to operate interventions aimed at assisting health professionals and reducing maladaptive outcomes.

## Data Availability Statement

The raw data supporting the conclusions of this article will be made available by the authors, without undue reservation.

## Ethics Statement

The studies involving human participants were reviewed and approved by Ethical committee of the Department of Cognitive Sciences, Psychology, Educational and Cultural Studies (COSPECS), University of Messina, Italy Ethical committee number: COSPECS_11_2020. The patients/participants provided their written informed consent to participate in this study.

## Author Contributions

EM made a significant contribution to design the research study, draft the manuscript, revised it critically, also performing the statistical analysis, and providing the interpretation of data. IM and AS made a significant contribution to design and revise the research study. SS gave the final approval. All authors listed have made a substantial, direct and intellectual contribution to the work, and approved it for publication.

## Conflict of Interest

The authors declare that the research was conducted in the absence of any commercial or financial relationships that could be construed as a potential conflict of interest.
